# Association Between Type D Personality and Cardiovascular Disease History: Cross-Sectional Study

**DOI:** 10.2196/79159

**Published:** 2026-03-10

**Authors:** Keren Grinberg, Yael Sela

**Affiliations:** 1 Nursing Sciences Department, Faculty of Social and Community Sciences, Ruppin Academic Center, Emek Hefer, 4025000, Israel, 972-9-8981056, 972-9-8983056

**Keywords:** type D personality, cardiovascular disease, psychological distress, depression, anxiety, stress

## Abstract

**Background:**

Type D personality, characterized by high negative affectivity and social inhibition, has been linked to poorer mental health and heightened risk for adverse cardiovascular outcomes. Although previous studies have examined associations between type D personality, psychological distress, and cardiovascular disease (CVD), many have assessed these factors independently, relied on clinical samples, or overlooked the simultaneous assessment of psychological distress and CVD history. Consequently, less is known about how type D traits relate to emotional distress and CVD history within the general population. Understanding these relationships may support early identification of at-risk individuals and strengthen the integration of psychological screening into cardiovascular care.

**Objective:**

This study aimed to (1) examine associations between type D personality, emotional distress (depression, anxiety, and stress), and self-reported CVD history; (2) compare distress levels among participants with and without CVD history; and (3) determine whether type D personality predicts emotional distress independent of demographic factors and CVD history.

**Methods:**

A cross-sectional online survey was completed by 146 adults aged 30 to 85 years, recruited through convenience and snowball sampling on social media. Type D personality was assessed using the Type D Scale–14, and emotional distress was measured using the Depression Anxiety and Stress Scale-21 items. CVD history was captured through a single self-report question regarding prior diagnosis of a cardiovascular condition. Descriptive statistics characterized the sample. Two-tailed independent samples *t* tests compared distress between individuals with and without type D personality and between participants with and without CVD history. Pearson correlation coefficients examined associations among key variables. Hierarchical multiple regression assessed whether type D personality predicted emotional distress beyond age, gender, education, and CVD history.

**Results:**

Of the 146 participants, 40 (27.4%) reported a history of CVD and 62 (42.5%) met criteria for type D personality. Individuals with type D personality exhibited significantly higher depression, anxiety, and stress levels than non–type D participants (all *P*<.001). Participants with CVD history also reported greater distress compared with those without CVD history. Hierarchical regression analyses showed that type D personality remained a strong independent predictor of emotional distress (*β*=.46; *P*<.001) after adjusting for demographics and CVD history. CVD history made an additional but smaller contribution to distress (*β*=.18; *P*=.008). These findings highlight the cumulative influence of personality traits and cardiovascular background on psychological well-being.

**Conclusions:**

Type D personality traits have been associated with higher levels of psychological distress and with a greater likelihood of self-reported CVD history in the general population. Type D personality remained a significant predictor of distress after accounting for demographic factors and cardiovascular history, underscoring its potential role in early psychological risk identification. Incorporating brief personality and mental health screening into cardiovascular assessment may support more comprehensive care.

## Introduction

### Background and Rationale

Cardiovascular diseases (CVDs) remain the leading cause of mortality worldwide, resulting from a complex interplay of genetic, physiological, behavioral, and psychosocial factors [1,2]. While traditional risk factors such as age, hypertension, smoking, and dyslipidemia are well established, there is growing recognition of the contribution of psychological characteristics to cardiovascular risk and prognosis [3,4]. Among these, type D (“distressed”) personality has drawn attention due to its association with poor mental health and adverse cardiac outcomes [5].

Type D personality is defined by the joint presence of negative affectivity (NA), the tendency to experience negative emotions, and social inhibition (SI), the tendency to suppress emotional expression and avoid social interactions [6]. Individuals with this personality profile are more likely to report symptoms of depression, anxiety, and stress and to experience low social support, poor quality of life, and unfavorable health behaviors [7,8]. This personality pattern is considered relatively stable over time and is found in both clinical and nonclinical populations [9]. Habibović et al [10] found that among patients with CVD, type D personality might be associated with lower social engagement, which could, in turn, partly explain its association with adverse health outcomes.

Emerging evidence suggests that type D personality is associated with an increased risk of cardiovascular morbidity and mortality, independent of traditional risk factors. Proposed mechanisms include heightened physiological stress responses (eg, hypothalamic-pituitary-adrenal axis dysregulation and inflammation), maladaptive coping strategies, and delayed health-seeking behavior [11,12]. Moreover, type D personality may interact synergistically with emotional distress to compound cardiovascular risk [13,14].

Despite increasing interest in this construct, several gaps remain. First, many studies have focused on patients with known cardiac diagnoses, limiting generalizability to broader populations. Second, type D personality and emotional distress (eg, depression, anxiety, and stress) are often examined in isolation, rather than concurrently, despite their theoretical and empirical overlap. Third, there is a lack of studies conducted in non-Western or diverse sociocultural contexts, such as Israel, where demographic and psychosocial profiles may differ.

This study addresses these gaps by simultaneously examining type D personality, psychological distress, and self-reported CVD history in a community sample. Unlike prior work focusing exclusively on clinical end points, our goal is to characterize the psychological profile of adults with and without cardiac history to assess whether type D personality is associated with greater emotional distress and cardiovascular vulnerability.

### Objectives and Hypotheses

This study has two primary aims: (1) to examine whether individuals with type D personality report higher levels of depression, anxiety, and stress compared to non–type D individuals; and (2) to assess whether type D personality is an independent predictor of psychological distress after controlling for age, gender, education, and cardiovascular history.

On the basis of prior literature and theoretical models, we hypothesize the following:

- Hypothesis 1—individuals with type D personality will report significantly higher emotional distress (depression, anxiety, and stress) than those without type D traits.
- Hypothesis 2—type D personality will significantly predict psychological distress after adjusting for demographic and medical variables.

By testing these hypotheses, this study aimed to contribute to a more nuanced understanding of how personality traits relate to psychological well-being and cardiovascular history. Such knowledge may help inform early screening and targeted interventions in cardiac care settings.

## Methods

### Study Design

A cross-sectional, quantitative study was conducted to assess associations between type D personality, psychological distress, and self-reported cardiovascular history in a nonclinical adult sample. The study was part of a larger survey-based investigation examining personality and health factors.

### Participants and Recruitment

A total of 146 participants were recruited via convenience and snowball sampling using targeted advertisements on Facebook and WhatsApp (Meta Platforms, Inc) between January and April 2022. Inclusion criteria included being aged 30 years or older, fluency in Hebrew, and access to the internet. Participants completed an anonymous online questionnaire hosted on Google Forms (Multimedia Appendix 1). Sensitivity analysis confirmed that the sample size provided sufficient statistical power for the logistic and linear regression models used.

### Ethical Considerations

The study was approved by the Institutional Review Board of Ruppin Academic Center (approval code 251-L/22). Informed consent was obtained electronically from all participants before participation. All responses were anonymous. Participants received no monetary compensation. All data were deidentified before analysis.

### Measures

#### Demographics and Health History

A background questionnaire collected data on age, gender, marital status, number of children, education level, religious identification, and self-reported cardiovascular history. CVD history was determined via the following question: “Have you ever been diagnosed with a cardiovascular condition or experienced a cardiac event (Yes/No)”?

#### Type D Personality

The Type D Scale–14 [8] was used to measure type D personality. The scale includes 14 items rated on a 5-point Likert scale, yielding 2 subscales: NA and SI. Participants scoring 10 or greater on both subscales were classified as type D. The scale has demonstrated good reliability in Hebrew-speaking populations (α>.80).

#### Psychological Distress

The Depression Anxiety Stress Scale (DASS)-21 items [13] was used to assess emotional distress. The 21 items measure depression, anxiety, and stress over the previous week using a 4-point Likert scale. Subscale scores were calculated and multiplied by 2 to match standard interpretations. Internal consistency was high (α=.87-.91).

### Data Screening and Statistical Analyses

Data were screened for completeness, normality, and outliers. All variables met the assumptions for parametric testing. Descriptive statistics were computed for all variables.

Independent samples *t* tests and chi-square tests compared psychological outcomes and type D classification across demographic groups and CVD history. Pearson correlations examined associations among continuous variables.

Hierarchical linear regression was used to evaluate whether type D personality predicted psychological distress (total DASS score), after controlling for age, gender, education, and CVD history. All analyses were conducted using SPSS (version 28; IBM Corp). Two-tailed independent samples *t* tests with *P*<.05 were considered significant. Effect sizes (ie, Cohen *d*, η², and *R*²) were reported.

## Results

### Sample Characteristics

Of the 146 participants, 40 (27.4%) reported a history of CVD. The sample was predominantly Jewish (140/146, 95.9%), secular (102/146, 70%), and highly educated (116/146, 80% held postsecondary degrees). Table 1 presents detailed demographic and psychological characteristics. Participants were categorized into 6 age groups: 30 to 39, 40 to 49, 50 to 59, 60 to 69, 70 to 79, and 80 to 85 years. The age distribution indicated that the majority of participants (95/146, 65%) were aged 50 to 70 years, while an additional 20% (29/146) were aged 70 to 85 years. Younger participants were less represented, with 5% (8/146) aged 30 to 39 years and 10% (15/146) aged 40 to 49 years. In total, 66% (97/146) of participants were female.

**Table 1.** Descriptive statistics and internal consistency of psychological measures among Israeli adults (N=146).

Variable	Values, mean (SD)		Cronbach α
Negative affectivity	11.4 (6.7)		0.89
Social inhibition	13.2 (6.1)		0.92
Depression	14.6 (5.8)		0.87
Anxiety	12.7 (5.4)		0.89
Stress	15.9 (6.5)		0.91
Distress (total)	43.2 (14.9)		0.94

### Prevalence of Type D Personality

On the basis of the Type D Scale–14, 42% (62/146) of participants met the criteria for type D personality . Type D participants were more likely to report a history of CVD (54/146, 36.9%) than non–type D participants (29/146, 20.2%; *χ*²_1_=4.5; *P*=.03).

### Group Differences in Psychological Distress

Participants with type D personality scored significantly higher on depression (mean 19.2, SD 5.0) than non–type D participants (mean 11.3, SD 4.5; *P*<.001; Cohen *d*=1.72), indicating a large effect size. Similarly, anxiety (mean 16.5 vs 9.7, SD 4.6 vs 3.9; *P*<.001; Cohen *d*=1.63) and stress (mean 21.0 vs 12.1, SD 5.3 vs 4.8; *P*<.001; Cohen *d*=1.76) were markedly higher in type D participants, reflecting strong group differences. Psychological distress differed between the study groups, as shown in Table 2.

**Table 2.** Group differences in depression, anxiety, and stress between participants with and without type D personality, based on summed Depression Anxiety Stress Scale–21 items subscale scores (range 0‐21; N=146).

Variable	Type D (n=62), mean (SD)	Non–type D (n=84), mean (SD)	*t* value (*df*)	*P* value
Depression	19.2 (5.0)	11.3 (4.5)	8.12 (144)	<.001
Anxiety	16.5 (4.6)	9.7 (3.9)	7.89 (144)	<.001
Stress	21.0 (5.3)	12.1 (4.8)	8.48 (144)	<.001

### Associations With Cardiovascular History

Participants with CVD history had higher mean type D scores (mean 1.42, SD 0.61) and higher levels of psychological distress than those without CVD. Depression, anxiety, and stress were significantly elevated among those with CVD (all *P*<.05).

### Regression Analysis

A 2-step hierarchical regression evaluated predictors of psychological distress. Step 1 included demographics and CVD history and explained 14.6% of the variance (*F*_4,141_=6.05; *P*<.001). Step 2 added type D personality and accounted for an additional 15.1% of variance (Δ*F*=29.64; *P*<.001), with the final model explaining 29.7% of total variance (*F*_5,140_=14.87; *P*<.001).

Type D personality was the strongest predictor (*β*=.46; *P*<.001), followed by CVD history (*β*=.18; *P*=.008). Age, gender, and education were not significant.

## Discussion

### Principal Findings

This study found that type D personality was strongly associated with elevated depression, anxiety, and stress in a community-based Israeli sample, with effects persisting after adjustment for age, gender, education, and CVD history. Participants with CVD also had higher type D scores and distress, although personality traits showed a stronger effect. The prevalence of type D (62/146, 42%) exceeded global community estimates, potentially reflecting cultural or contextual factors. This unusually high prevalence may reflect specific cultural, societal, or psychosocial characteristics within the Israeli context, such as collectivist norms, social stressors, or health awareness, highlighting the importance of considering local sociocultural factors when interpreting type D personality prevalence and its psychological correlates. These results align with epidemiologic evidence showing shifts in cardiovascular determinants and the growing role of psychosocial risk factors [15-17], as well as updates highlighting stress pathways in CVD pathophysiology [16]. Depression levels in participants with type D personality were in the moderate range (mean 19.2, SD 5.0), consistent with literature linking depressive symptoms to increased cardiac risk via behavioral, autonomic, inflammatory, and platelet activation mechanisms [18,19]. Such clustering of distress in individuals with type D personality mirrors psychocardiology findings that integrate psychological traits with cardiovascular biology and epidemiology [20].

By jointly examining type D personality, psychological distress, and CVD history, this study extends previous work focused mainly on clinical cardiac populations, demonstrating similar associations in nonclinical adults. Mechanistically, high NA and SI may limit social support, encourage maladaptive coping, and promote biological dysregulation, all of which can contribute to cardiovascular vulnerability [16,18]. The findings suggest potential value in community and primary care screening for type D traits and distress, in line with clinical reviews emphasizing early psychosocial intervention to improve cardiac outcomes [19,21]. Although the cross-sectional design limits causal inference, and the sample’s demographic profile may affect generalizability, these results support a multifactorial model where stable personality traits, affective states, and cardiovascular history interact within the broader epidemiologic trends in CVD [15-17,20].

It should be noted that because type D personality includes NA and the outcome measure (DASS-21) assesses depression, anxiety, and stress—all facets of negative affect—our analysis may partly reflect conceptual overlap rather than an independent predictive effect, highlighting a tautological limitation that warrants careful interpretation.

### Limitations

Several limitations warrant discussion. First, the use of self-reported CVD history introduces potential misclassification and recall bias. Although we included examples in the survey question, objective verification (eg, medical records) was not feasible. Additionally, the use of a single self-report item for CVD history limits granularity and may affect associations with type D personality and psychological distress.

Second, the cross-sectional design precludes causal inference. While we propose that type D traits may precede emotional distress, it is also plausible that chronic distress shapes personality expression (reverse causality). Future longitudinal research is needed to disentangle these pathways. In addition, the convenience and snowball sampling may have introduced self-selection bias, as individuals with greater psychological concerns or distress may have been more likely to participate, possibly inflating type D prevalence and its association with distress.

Third, the sample was predominantly female, Jewish (140/146, 95.9%), highly educated, and secular. This demographic specificity, along with the nonrandom recruitment strategy, limits the generalizability of the findings. The prevalence of type D personality in our sample (62/146, 42%) was higher than typically reported in community-based studies, which may reflect characteristics of the recruited population and further limits external validity.

Fourth, the overlap between type D (particularly the NA subscale) and general negative affect raises concerns about multicollinearity in statistical models. Future studies should consider examining subscale interactions or partial correlations to clarify unique contributions.

Fifth, our analyses did not account for several established cardiovascular risk factors, including behavioral factors (eg, smoking, physical activity, and diet), biomedical factors (eg, BMI, diabetes, and dyslipidemia), and broader socioeconomic variables (eg, income and employment status). These unmeasured variables may confound the observed associations between type D personality, psychological distress, and CVD history and should be considered in future research.

Future research should use longitudinal and multimethod designs to evaluate the causal relationships among personality traits, emotional distress, and cardiovascular outcomes. Clinical studies involving medically verified diagnoses, as well as diverse cultural and demographic samples, are needed to enhance external validity. In addition, the development and validation of brief screening tools for type D personality in primary care and cardiology settings could facilitate early identification. Intervention studies should examine whether reducing emotional distress in individuals with type D personality leads to improvements in cardiovascular outcomes.

### Recommendations

Future studies should further explore the clinical implications of incorporating psychological screening, such as type D personality, into cardiovascular care. Additional research is warranted to evaluate how integrating psychological assessment into routine practice may contribute to more personalized prevention and intervention strategies.

### Conclusions

This study demonstrates that type D personality is significantly associated with elevated psychological distress and a history of CVD, even within a community sample. These findings highlight the importance of considering psychological factors when examining cardiovascular health outcomes.

## Supplementary material

10.2196/79159 Multimedia Appendix 1 Survey of the study (in Hebrew).

## Abbreviations

CVD: cardiovascular disease
DASS-21: Depression Anxiety Stress Scales
NA: negative affectivity
SI: social inhibition
