# Supplementary material for: Association Between Type D Personality and Cardiovascular Disease History: Cross-Sectional Study
Source: JMIR Cardio. 2026 Mar 10;10:e79159. doi: 10.2196/79159 (PMC12974995; doi:10.2196/79159)
Supplement: Multimedia Appendix 1 [file cardio-v10-e79159-s001.docx]

הקשר בין סוג אישיות D ומידת הדחק לבין מחלות/אירועי לב בקרב חולים קרדיאליים

**חלק א' – פרטים דמוגרפיים**

אנא מלא.י את השאלון. יש לבחור בתשובה אחת לכל שאלה.

**מין**

- זכר
- נקבה

**גיל**

- **30-39**
- **40-49**
- **50-59**
- **60-69**
- **70-79**
- **80-85**

**מצב משפחתי**

- רווק.ה
- נשוי.אה
- גרוש.ה
- פרוד.ה
- ברית זוגית
- חד הורי.ת

**האם יש לך ילדים?**

- כן
- לא

**דת**

- יהודי.ה
- נוצרי.ה
- מוסלמי.ת
- דרוזי.ת
- חסר דת
- אחר

**כיצד היית מגדיר.ה את עצמך ?**

- חילוני.ת
- דתי.ה
- מסורתי.ת
- חרדי.ת
- אתאיסט.ית
- אחר

**רמת השכלה**

- תיכונית או על תיכונית
- תואר ראשון
- תואר שני
- תואר שלישי ומעלה

**חלק ב' - רקע קרדיאלי**

יש לבחור בתשובה אחת בלבד. יש לשים לב כי רקע קרדיאלי (לבבי) הכוונה לכל מחלה/הפרעה לבבית וכלי דם, בכל חומרה שהיא, אשר מאובחנת בקופת החולים.

**האם את.ה בעל.ת רקע רפואי לבבי ?**

- כן
- לא

**חלק ג' – שאלון אישיות**

**שאלון העוסק בדפוסי אישיות**

**ההצהרות המופיעות מטה משמשות לעיתים תכופות אנשים בכדי לתאר את עצמם. אנא קרא.י כל אחת מההצהרות וסמן.י את התיבה המתאימה ביותר לתאר את מידת הסכמתך עם ההצהרה. אין תשובה נכונה או לא נכונה, השקפתך האישית היא שחשובה.**

|  | בכלל לא | במידה מועטה | במידה בינונית | במידה רבה | במידה רבה ביותר |
| --- | --- | --- | --- | --- | --- |
| אני יוצר/ת קשר בקלות כשאני פוגש/ת אנשים |  |  |  |  |  |
| לעתים קרובות אני עושה עניין מדברים לא חשובים |  |  |  |  |  |
| לעתים קרובות אני מדבר/ת עם זרים |  |  |  |  |  |
| לעתים קרובות אני מרגיש/ה אומלל/ה |  |  |  |  |  |
| לעתים קרובות אני נרגז/ת |  |  |  |  |  |
| לעתים קרובות אני מרגיש/ה לא משוחרר במהלך אינטראקציות חברתיות |  |  |  |  |  |
| אני מתבונן/ת בעגמומיות על דברים |  |  |  |  |  |
| אני מתקשה בפתיחה בשיחה |  |  |  |  |  |
| יש לי מצב רוח רע לעתים קרובות |  |  |  |  |  |
| אני אדם מסוגר |  |  |  |  |  |
| אני מעדיף/ה לשמור על ריחוק מאנשים |  |  |  |  |  |
| אני מוצא/ת את עצמי לעתים קרובות מוטרד/ת |  |  |  |  |  |
| לעתים קרובות אני מדוכא/ת |  |  |  |  |  |
| אני לא מוצא/ת נושאים נכונים לשיחה כשאני בחברה |  |  |  |  |  |

**חלק ד' – שאלון DASS-21**

**שאלון העוסק במידת חרדה, דיכאון ולחץ.**

יש לקרוא כל אמירה ולסמן במספרים 0-3 עד כמה האמירה מתארת את מצבך *במהלך השבוע האחרון*. שים.י לב, כי אין תשובה נכונה או שגויה. אין צורך להתעכב על כל אמירה יתר על המידה. 0- האמירה לא מתארת את מצבי כלל. 1- האמירה מתארת את מצבי באופן חלקי, או בחלק מהזמן. 2- האמירה מתארת את מצבי באופן ניכר או בחלק ניכר מהזמן. 3- האמירה מתארת את מצבי מאוד, או ברוב הזמן.

|  | 0 | 1 | 2 | 3 |
| --- | --- | --- | --- | --- |
| התקשיתי להיות נינוח/ה |  |  |  |  |
| חשתי ביובש בפה |  |  |  |  |
| התקשיתי לחוות כל הרגשה חיובית |  |  |  |  |
| חשתי קוצר בנשימה (לדוגמה, נשימה מואצת במיוחד, חוסר אוויר בהיעדר פעילות גופנית מאומצת) |  |  |  |  |
| לא היה לי הכוחות לעשות דברים |  |  |  |  |
| נטיתי להגזים בתגובותיי למצבים מסוימים |  |  |  |  |
| חשתי רעד (למשל, בידיים) |  |  |  |  |
| הרגשתי שאני מתעצבן/ת יותר מידי |  |  |  |  |
| חששתי ממצבים בהם אולי אכנס לחרדה ואעשה צחוק מעצמי |  |  |  |  |
| הרגשתי שאין לי למה לצפות בחיים |  |  |  |  |
| הרגשתי שאני קצר/ת רוח |  |  |  |  |
| התקשיתי להירגע |  |  |  |  |
| חוויתי דכדוך ותחושות עצבות |  |  |  |  |
| הייתי חסר/ת סובלנות כלפי כל דבר שהפריע לי במעשיי |  |  |  |  |
| הרגשתי שאני קרוב/ה למצב של פאניקה |  |  |  |  |
| לא הצלחתי להתלהב משום דבר |  |  |  |  |
| הערכתי העצמית כאדם הייתה מאוד נמוכה |  |  |  |  |
| הרגשתי רגיש/ה ופגיע/ה למדי |  |  |  |  |
| חשתי בפעילות ליבי גם ללא פעילות גופנית (לדוגמה, הרגשת עלייה בקצב הלב, החסרת פעימת לב) |  |  |  |  |
| הייתי מפוחד/ת גם ללא סיבה מיוחדת |  |  |  |  |
| הרגשתי שהחיים חסרי משמעות |  |  |  |  |
